# The largest vaccination campaign in history: A golden opportunity for bundling public health interventions

**DOI:** 10.7189/jogh.11.03076

**Published:** 2021-05-22

**Authors:** Moosa Tatar, Fernando A Wilson

**Affiliations:** 1 Matheson Center for Health Care Studies, University of Utah, Salt Lake City, Utah, USA; 2 Department of Population Health Sciences, University of Utah, Salt Lake City, Utah, USA; 3 Department of Economics, University of Utah, Salt Lake City, Utah, USA

One year after the World Health Organization (WHO) declared the COVID-19 outbreak a global pandemic in March 2020 [1], more than 110 million COVID-19 cases, and two and half million deaths COVID-19 deaths were reported globally by the end of February 2021 [2]. The global efforts of governments, organizations, and private firms in developing vaccines were successful, and various vaccines (eg, Pfizer and BioNTech, Moderna, and Sinopharm) are being mass-produced and globally distributed. By the end of February 2021, more than 250 million people have been vaccinated against the COVID-19 virus in the world [3]. However, billions of people have not received the COVID-19 vaccination, and the biggest vaccination campaign in history will be ongoing for the foreseeable future.

Logistical planning and distribution of the COVID-19 vaccines is a multi-national and multi-organizational campaign and mostly coordinated by the COVAX initiative, which is signed by several countries (eg, United States, Germany, and Japan). The COVAX initiative led by the WHO, Gavi, the Vaccine Alliance, the Coalition for Epidemic Preparedness and Innovations (CEPI), and the United Nations Children's Fund (UNICEF) aims for fair and equitable COVID-19 vaccine distribution, especially to low to middle-income countries. In addition to mitigating the spread of COVID-19, this campaign is a golden opportunity to advance a number of critical public health initiatives including, for example, administration of underutilized vaccines (ie, Hepatitis B (HepB), Cholera, and HPV vaccine), rapid screening for infectious disease (eg, Hepatitis C (HepC), HIV, and Ebola), and distributing supplies or educational materials (eg, brochures, birth control). No patients were involved in this research, and the article does not involve human participants or personal medical information.

Currently, the Centers for Disease Control and Prevention (CDC) recommends new or underutilized vaccines to be included in the routine childhood immunization schedule (eg, HepB), Haemophilus influenza type b (Hib) vaccine) and for older or at-risk populations (eg, the cholera and human papillomavirus (HPV), and meningococcal vaccines). “Piggy-backing” on the worldwide COVID-19 vaccination campaign might also be a unique and opportune time to promote new and underutilized vaccines and, thus, make great strides toward mitigating these infectious diseases and improving population health.

For example, tuberculosis (TB) is a preventable disease and often curable; however, it is a leading cause of death worldwide. Further, undiagnosed and untreated TB often leads to permanent and debilitating conditions such as loss of lung function [4]. Hundreds of millions of individuals, especially in developing (ie, low- and middle-income) countries have latent TB infection, and, of these, ten million people develop active TB that infects an average of ten other people each year [5]. Unfortunately, the COVID-19 pandemic has substantially disrupted efforts to monitor and mitigate TB and other infectious diseases and public health resources were re-allocated away from many of these efforts to combat COVID-19 [6].

Similar to TB infection, there is a long-term public health challenge in addressing rates of chronic HepC infection. Globally, there are more than 70 million individuals infected with HepC, and, within the United States, between 2 and 3.5 million people have chronic HepC infection, and the majority of them are not aware of their disease. Consequently, undiagnosed HepC often results in permanent and severe liver impairment and premature death, and thus early screening for HepC is critical. Studies suggest undertaking targeted HepC screening for at-risk people [7], and the CDC and the US Preventive Services Task Force (USPSTF) promote universal HepC screening for all adults aged 18 and older in the United States [8,9]. Because HepC is often spread via injectable illicit drug use, it is possible that we may experience increasing rates of HepC due to evidence of increasing drug use since the beginning of the COVID-19 pandemic [10].

The COVID-19 vaccination campaign creates a unique opportunity for screening and early diagnosis for these and other long-standing public health threats such as HIV or diabetes. Tuberculosis, HepC, and HIV are co-epidemics and, therefore, pairing COVID-19 vaccinations with the opportunity to provide additional screening and diagnoses will have an enhanced, cost-effective impact on population health, particularly in low to middle-income countries which are resource constrained. These screenings also require additional time and resources including, for example, counseling time and associated costs. A rapid antigen or oral fluid antibody test for HIV involves either a finger prick or an oral fluid sample, and they can provide results in 20 to 30 minutes. A TB blood test or a HepC Virus antibody test requires a blood sample, and public health or medical professionals would need to follow-up with individuals concerning their test results. Other screenings which are more involved or require patient fasting or a follow-up visit such as the tuberculin skin test or A1C test for blood glucose levels can be offered to individuals when scheduling their COVID-19 vaccinations.

**Figure:**
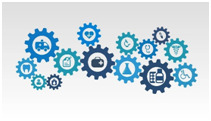
Photo: From https://pixabay.com/illustrations/health-care-medicine-healthy-2082630/.

In addition to public health screenings, COVID-19 vaccination sites are ideal in distributing educational materials to inform patients on the above infectious and chronic diseases and can help them identify symptoms or refer them to additional health resources. These materials can be bundled as a Preventive Health Toolkit, increasing patient awareness of symptoms and available resources relating to infectious, sexually transmitted, and chronic diseases as well as mental health status (eg, anxiety, depression), domestic violence, and other public health concerns. The Toolkit could be in the form of a brochure or CD, or even in the form of health supplies such as birth control. Patient contact information can also be solicited and used to follow-up after testing and distribute targeted educational materials and resources via mail or email.

This is a unique and valuable opportunity to advance public health across a range of initiatives and to improve public trust in the health care system and fight against health science misinformation, not only regarding COVID-19, but with TB, HepC, HIV and other infectious and chronic diseases. Successful global mitigation of the COVID-19 virus in conjunction with tackling other infectious and chronic diseases, along with increasing public awareness and information on individual and public health challenges, has the potential to make great strides toward advancing population health and decreasing global disparities in health outcomes. However, in addition to funding and resources, these interventions will require logistical planning (ie, storage requirements) and have other challenges (eg, surveillance, safety, credibility, etc.) to increase their reach and effectiveness. Also, coordinating these interventions needs global commitment and efforts (possibly by Global Health Initiatives, WHO, UNESCO, COVAX, and World Bank).
